# Local transplantation, adaptation, and creation of AI models for public health policy

**DOI:** 10.3389/frai.2023.1085671

**Published:** 2023-08-16

**Authors:** Eleonore Fournier-Tombs

**Affiliations:** ^1^Anticipatory Action and Innovation, United Nations University Centre for Policy Research, New York, NY, United States; ^2^Civil Law Faculty, University of Ottawa, Ottawa, ON, Canada

**Keywords:** artificial intelligence, modeling, public health, localization, policy

## Abstract

This paper presents the Transplantation, Adaptation and Creation (TAC) framework, a method for assessing the localization of different elements of an AI system. This framework is applied in the public health context, notably to different types of models that were used during the COVID-19 pandemic. The framework aims to guide AI for public health developers and public health officials in conceptualizing model localization. The paper provides guidance justifying the importance of model localization, within a broader context of policy models, geopolitics and decolonization. It also suggests procedures for moving between the different elements in the framework, for example going from transplantation to adapation, and from adaptation to creation. This paper is submitted as part of a special research topic entitled: A digitally-enabled, science-based global pandemic preparedness and response scheme: how ready are we for the next pandemic?

## 1. Introduction

The COVID-19 pandemic has seen a rise in the uses of predictive analytics in public health policy. Many epidemiological techniques have been adapted to incorporate new machine learning methods, allowing for more granular predictions. In turn, these predictions have been used to inform public health measures and humanitarian interventions globally. From the early days of the pandemic, models were made open source and adapted as necessary to regional contexts (UNOCHA, 2021). This was done largely through the “swapping out” of training datasets, but also, occasionally, in the tuning or even modification of parameters.

Irrelevant data, improper development and calibration of models can have important consequences, not only in terms of over or under predicting disease spread, but also in inappropriate uses, ignoring local contexts. This phenomenon has been observed in artificial intelligence (AI) modeling for public health policy, but also in many other types of models which are developed in the Global North and then implemented in countries in the Global South (Cooke, 2003). More specifically, a part of it has been designated as ≪data colonialism≫ (Couldry and Mejias, 2018), ≪data universalism≫ (Milan and Treré, 2019), or ≪digital universalism≫, which raise concerns about power asymmetries (Ricaurte, 2019), notably in terms of the flow of technologies from North to South, and the flow of data and resources from South to North. In this sense, AI models can have important geopolitical repercussions and risk exacerbating inequalities (Luengo-Oroz et al., 2021).

We therefore propose a way of conceptualizing these localization efforts, drawing on existing modeling practices in public health. We present a modeling localization matrix, the TAC framework (Transplantation—Adaptation—Creation) which aims to guide the analysis of AI models for public health.

In order to illustrate this framework, we provide examples of public health models developed in response to the COVID-19 pandemic and evaluate them using the framework, discussing the applicability and reliability of different dimensions of TAC. Throughout the paper, we discuss the context of geopolitical dynamics in public health modeling, which demonstrates a phenomenon also present in economic, legal (Merry, 1991) and other technological models (Sahbaz, 2019). Legal models, or legal systems, for example, have also been exported to the Global South during colonization, and are today criticized by a political and intellectual movement toward decolonizing law (Foley, 2022). Our hope is not only to provide guidance to practitioners in evaluating and developing public health models in future epidemics, but also to raise awareness to a broader conceptual issue with policy-informing models and legal frameworks and their adaptation from one country to the next.

## 2. The use of AI in public health

In this paper, we focus on the use of AI for public health policy. AI technologies are used widely in a variety of sectors, and the applicability of the TAC framework to these sectors will be discussed further in the conclusion. However, in the interest of developing a policy-relevant conceptual analysis, we will concentrate our examples of AI in the public health policy domain. Additionally, this sector has seen a unique rise in AI activity, notably due to the COVID-19 pandemic, which accelerated the digital transformation of public health. It is with this in mind that we begin to explore the current uses of AI in public health policy.

Public health policy involves decisions, plans and actions aimed at achieving certain health objectives in a population. These objectives can be long-term, such as cancer prevention or obesity reduction, or shorter-term, such as curbing an epidemic. Generally, public health also has systemic objectives, such as avoiding the over-burdening of a public hospital system or reducing inequality and exclusion in healthcare access.

Given this context, there are several ways in which AI technologies can be used to support public health policies, notably planning of health services, communicating to the public, and tracking infections, as summarized in Table 1. We will explore each one in turn.

**Table 1:** Common uses of AI models for public health policy.

**Activity**	**Description**
Health services planning	Machine learning tools incorporated into epidemiological models to predict the severity and spread of disease, in order to better plan public health interventions.
Communicating to the public	Chatbots using Natural Language Processing (NLP) techniques are used to provide public health recommendations to the public
Tracking infections	Predictive tools incorporated into some contact tracing applications to predict possibility of infection between two parties or in a public area

### 2.1. Health services planning

Over the last few years, we have seen an increasing use of AI techniques, specifically machine learning, in epidemiological contexts. At the beginning of the COVID-19 pandemic, many of these models were released publicly, where researchers and public health agencies could modify the input data and settings to obtain their own predictions on the spread and severity of the virus. Certain models were also built at a global level, using publicly available administrative data. These models allowed public health agencies to select appropriate non-pharmaceutical measures to the pandemic, such as school closures, social distancing, and masking. They also allowed the agencies to anticipate hot spots and reach groups that might be at higher risk, such as the elderly or those with pre-existing conditions.

There were several advantages to these AI models. First, they served as a planning and communication tool, by allowing public health analysts to build scenarios which would allow policymakers to evaluate the positive and negative effects of certain measures. They could also be used to plan vaccine purchases, open new COVID-19 wards in hospitals, or plan other medical initiatives. The projections produced by the models were then shared with the public, to obtain their buy-in for certain particularly restrictive interventions, such as curfews. The projections also contained a time element, which could map out the effect at a given time of an intervention, and how long this intervention might last.

Using machine learning methods in epidemiology allowed, in principle, for the processing of much larger datasets, leading, potentially, to higher accuracy rates.

During the pandemic, Imperial College developed a suite of COVID-19 planning tools which aimed to inform the work of policy makers globally (Imperial College, 2022). These tools included the MRIIDS (Mapping the Risk of International Infectious Disease Spread), which also was used for the Ebola virus, as well as a COVID-19 Orphanhood Calculator. Many other organizations and researchers shared similar tools, such as BeCaked, which used explainable AI (Nguyen et al., 2022), and a University of Waterloo model predicting COVID-19 risk in patients (University of Waterloo, 2022). Regional models have been developed as well. Kong led the development of an AI tool focused on making predictions for Africa (York, 2021). Han used AI to model the spread of the disease in animals in Southeast Asia and Central America (Geddes, 2022).

There have also been critics of the usefulness of certain AI models for COVID-19 modeling. In an article for the MIT Technology Review, Heaven argued that “*In the end, many hundreds of predictive tools were developed. None of them made a real difference, and some were potentially harmful*” (Heaven, 2022). Several pitfalls have been identified by researchers, including using the same, flawed datasets across many of these models, not properly selecting parameters, and failing to remove duplicate data (Roberts et al., 2021).

### 2.2. Communicating to the public

A key component of public health policy in an epidemic context is communicating to the public. Communicable diseases that drive epi- and pandemics are largely governed by individual behaviors, such as spending time with others, touching, handwashing, wearing protective masks, and so on. An important consideration for public health agencies in this context is therefore accessing members of the public. This has been done, historically, in a variety of ways, from dropping flyers, to radio broadcasts, to public health websites. An important AI application in this domain was the use of the chatbot, which allowed for communications both related to prevention and to treatment. Chatbots have been particularly useful for their low cost, reaching people directly through social media applications, for example, without necessitating the participation of a medical practitioner. They are also often touted for their ability to facilitate basic health care services to marginalized groups, such as women (in places where women and girls have lower access to healthcare), people living in remote communities, and people with low literacy skills.

Chatbots primarily use the AI subfield of natural language processing (NLP), which allows models to interpret text and compose a response, usually by iteratively predicting the next word in a sentence. Chatbots used in public health typically use NLP models with a significant amount of non-AI rules added to them, such as pre-written responses, or decision-tree frameworks. This is because in this context, the text produced by the chatbot has to be as predictable as possible. Sometimes, NLP is used only in the interpretation of the patient or individual's request, the response being pre-written by a health practitioner, rather than composed by AI.

During COVID-19, a common user interface for public health chatbots was through the social media tool WhatsApp. A Senegalese account called Dr. COVID, no longer active today, shared public health information (Tworek et al., 2020). Coronabot, in Tunisia, was a chatbot available through Facebook that helped, according to its creators, more than 4500 families in remote areas of the country access public health information (Blaise, 2020). There was also LINE COVID, developed by Taiwan's Digital Minister Audrey Tang, which sent messages about which pharmacies had available mask supplies, using over 100 interactive maps developed by citizens (Timmerman, 2020).

### 2.3. Tracking infections

Finally, AI was used by many public health agencies to track individuals infected by the virus and warn those that they had met about possible contagion. Contact tracing applications were deployed around the world with a variety of features and data governance settings. In Canada, for example, an early proposal included behavioral analytics, which might predict the movements of infected people based on certain characteristics (Alsdurf et al., 2020). After public consultation, what was launched was much simpler, using Bluetooth to let people know if they had come within 2 m of an infected person (Gómez-Ramírez et al., 2021). Data entry was voluntary, and the government stored no personally identifiable information. However, in China, additional data sources and analytics were added, including possible tracking of a person's movements and credit card history in the time preceding the infection (Norton Rose Fulbright, 2020).

There have been many other uses of AI in medicine during the pandemic. For example, a Senegalese research team created a robot which used a number of AI technologies, including machine vision and NLP, to provide basic healthcare to patients in a Dakar hospital (Ollivier, 2020). AI to interpret medical images has also been used to diagnose COVID-19 and assess the risks of severe infection. This paper therefore focuses specifically on uses of AI specifically by public health authorities for public health planning or directive implementation.

## 3. Model localization in perspective

In the previous section, we discussed three types of AI models used in public health policy—machine learning-based forecasting, natural language processing, and behavioral analytics. However, the issue of localization, which we will further expound below, is not only relevant to AI models. In fact, the concept of a model, particularly as it informs policy, has a long and contentious history. In the following section, we will examine the issue of policy-informing models and localization in perspective, taking cues from other types of models, which have been used in development economics and law-making. We also examine issues related to the current geopolitics of AI, and how they have pushed AI development away from localization.

### 3.1. A very short history of models

In the following section, we explore the concept of models beyond AI, to illustrate broader historical and geopolitical trends which have influenced the way in which AI models have been disseminated globally. The notion of *model* has a long history in Western Civilization, calling to mind Plato's Theory of Forms, which proposes that the physical world is but a pale imitation of the world of ideas, or forms, which represent the non-physical essence of things. In modern definitions, the term *model* is used broadly to refer to an example which one should seek to emulate, as in the term *role model*. Even the profession of *model*, in which a person wears clothes that are available for sale, has become something to emulate, with professional models seen as representing an ideal of beauty.

In this context, the mathematical and technological concept of the model is more specifically described as: **“***a system of postulates, data, and inferences presented as a mathematical description of an entity or state of affairs*” (Merriam Webster, 2022). Mathematical, and by extension, computational models, allow for the codification of a certain set of data transformations, allowing calculations to be replicated in different contexts. Whether an abstract representation, or a physical *thing* (object, person or law), in our collective consciousness, models are seen as accurate, true, and even flawless. Conversely, antonyms for the word *model* include such terms as “*bad, low-grade, deficient, ordinary”*, and so on (Merriam Webster, 2022).

While the mathematical model draws on the development of a set of rules which can be abstracted from the application, the legal model can also be based on the notion of precedence, where often, the first legislation to address a certain societal issue becomes the model on which similar legislations in other jurisdictions are developed. The distinction in legal models can best be seen in the differences between civil law and common law, where civil law has its roots in a platonic abstract and general principle, while common law tends to be codified *post-facto*, by piecing together models from legislative decisions.

### 3.2. Economic, legal and health policy models

In the 1980s and 1990s, the International Monetary Fund (IMF) and the World Bank implemented Structural Adjustment Programs (SAPs) (Easterly, 2003) in many emerging economies—making its low-interest government loans conditional on austerity measures and restructuring of economies according to a template, developed by economists based in the United States. These initiatives were criticized for having a devastating impact on many developing country economies, arguably contributing to this day to low growth, poverty, and conflict (Ismi, 2004). Economies were encouraged to specialize, focusing on one or two core industries, and relying on trade agreements for the import of other goods (Battikha, 2002). Countries such as Senegal, turned to monocropping (of groundnuts, for example), which critics argued made them more dependent on the volatility of agricultural markets, and much more dependent on crop disease, pests, or natural disasters (Mbow et al., 2008).

Stiglitz (2002) writes in *Globalization and its Discontents* that the economic model used by the IMF during the 1980s and 1990s contributed to the 1997 Asian financial crisis, as well as low levels of development in Sub-Saharan Africa. These policies, encouraged by the organization to access development loans by poorer countries included fiscal austerity, high interest rates and trade liberalization. Shafiu (2021) argue that these impeded economic growth in Nigeria, while Stein (1992) documents a similar effect of IMF loan conditionality in Tanzania. Just like its African neighbors, Senegal found itself also negatively affected by these economic models, which were only set aside in the last decade (Dieye, 2020).

Beyond international banks, many former colonial powers continue to influence their former colonies, both through formal and informal ties. Both France and England, for example, maintain formal ties with their former colonies. France, arguably maintains macroeconomic power through the West and Central African Frank (CFA), which were pegged first to the French franc, and now to the euro (King, 2022), and England, through the Commonwealth system, which also arguably, allows it to maintain political influence in former British colonies and other countries, such as Rwanda and Togo.

France also has a system of *Territoires d'Outre Mer (TOM)* and maintains both formal and informal ties with former colonies outside of the TOM system as well, particularly in West Africa. Senegal is a priority country for France's foreign affairs strategy and participates in numerous yearly bilateral visits. For example, in 2017, as many as six bilateral visits took place, either in Dakar or in Paris, between high-level government officials, including the respective presidents [Ministère de l'Europe et des Affaires Étrangères (MEAE), Gouvernement Français, 2022].

However, geopolitical influence, to a certain extent, requires maintenance—hence the numerous visits. Legislative influence, on the other hand, can have a life of its own without much attention. One of the ways in which countries such as England and France maintained post-colonial influence was through the exportation of policy models. France, for example, was active in supporting its former colonies in West Africa to develop civil law-based legal systems, which were later criticized for failing to include cultural, tribal, and linguistic considerations. Both France and England also left an important educational heritage around the world, as did Spain. It is only in the last few years, for example, that Mexican states have included indigenous language in the public elementary school curriculum (Mazatlan Post, 2019).

It is therefore in this general context of model exportation from Global North countries that we examine the way in which artificial intelligence models can similarly be used to influence public policy, notably in the recent case of COVID-19 policies.

### 3.3. Geopolitics of AI

In artificial intelligence development, the largest powers are undoubtedly the United States and China. These countries both generate and receive the majority of international investment, even from neighboring countries. Canada, for example, spent millions of dollars on the development of a homegrown artificial intelligence start-up, Element AI, only to have it be bought up by the American company ServiceNow after a few years of operation (Silcoff, 2020). AI companies such as Palantir (Mitchell, 2016), Microsoft and Amazon (Zakrezwski, 2022) also spend millions of dollars annually on political lobbying activities, attempting to ensure that their interests are considered in legislation development.

The start-up purchasing of large American and Chinese companies and the lobbying activities are not the only ways in which these activities contribute to global inequality, however. Artificial intelligence companies are, by nature, user data harvesters. Much in the same way that former colonial powers have created a pathway for their own extractive companies to engage in natural resource mining in the Global South, they have also allowed their artificial intelligence companies to mine the data of Global South citizens. Today, 2.9 billion people globally use the Facebook application (Statista, 2022a), 1.4 billion use Instagram (Statista, 2022b), and 2.4 billion use WhatsApp (Statista, 2022c), all of which are owned by the American company Meta. Every day, the online activities, physical movements, and even discussions of each one of those users is recorded by Facebook. The data is then used internally to train its models, as well as sold to other model developers, such as advertisers and data brokers (Moore and Tambini, 2018).

Not only are financial resources related to AI concentrated in a handful of countries, but so are the modes of conceptualizing the AI itself. The majority of AI models are developed in these countries using training data that is largely dominated by their own culture, as well as developers that don't understand local contexts (Cook, 2020). While, as we have seen, more than a third of human beings use Facebook, the algorithms governing it, such as content and contact recommendation systems, facial recognition, and so on, were developed primarily using data that American developers had access to.

Over time, more and more biases have been discovered in relation to these types of models, from an inability to recognize the faces of persons of color (Buolamwini and Gebru, 2018), to discriminatory outputs for different groups (Fournier-Tombs and Castets-Renard, 2022), to exclusion of certain populations entirely (Park and Humphry, 2019). In a historical context of the geopolitics of models, this outcome is not entirely surprising. As we have seen, models of various kinds, whether they be economic, legal, or technological, have the potential to have a detrimental effect on country sovereignty and development when applied inappropriately.

### 3.4. Model localization

One of the most important solutions that has therefore been brought forward is that of model localization. In non-technological domains, this has taken the form of movements toward economic sovereignty, inclusion, or re-drafting of constitutions. When it comes to AI, there have been arguments that the most appropriate and empowering solutions should be local, which means decolonizing data (Calzati, 2021) and AI (Mohamed et al., 2020). This also echoes a trend toward digital sovereignty, which allows countries to manage their own digital infastructure and hardware, such as networks, storage, and microprocessors (Pohle and Thiel, 2021).

We note here that there is a stream in software development more broadly called localization, which is primarily concerned with interface translation. In this paper, we move toward a very different definition of localization, which involves thinking local at all stages of the AI development lifecycle. This conceptualization is aligned with inclusive models such as Arnstein's ladder for citizen participation, which examines citizen agency in projects, from manipulation to citizen control (Arnstein, 1969). Similarly, AI localization is about the autonomy and empowerment of those that are affected by the technologies, from conceptualization, to development, to governance and use.

In the figure below, we map the phases of the AI development lifecycle. We will return to these elements in the next part when we begin selecting indicators for model localization as they relate to the AI lifecycle.

A growing number of initiatives globally aim to put into practice some of these concepts of AI localization. For example, several projects have involved the promotion of AI development in Africa, including the Africa-Canada AI and Data Innovation Consortium, the AI Africa Consortium, and the African Observatory on Responsible Artificial Intelligence.

Given these principles of model localization the objective of the following section will be to provide a means of evaluating whether a model is appropriate for a given context. We detail the TAC Framework as a lens through which future model development can be assessed.

## 4. The TAC framework: transplantation, adaptation, and creation

As we have seen, the TAC Framework stands for Transplantation, Adaptation and Creation. Table 2 describes each of these three dimensions.

**Table 2:** Overview of TAC framework.

**Dimension**	**Description**
Transplantation	The model was developed in a high-income country primarily by developers who are not aware of how it will be applied in the target country. The technology is made available and those that use it are required to adapt.
Adaptation	The model was developed in a high-income country but is then adapted to the target country by developers who are aware of that country's context. This can be done by modifying the training data, the parameters, or the structure of the model itself.
Creation	The model is developed in the target country and is fully appropriate for the country's context.

**Figure 1:**
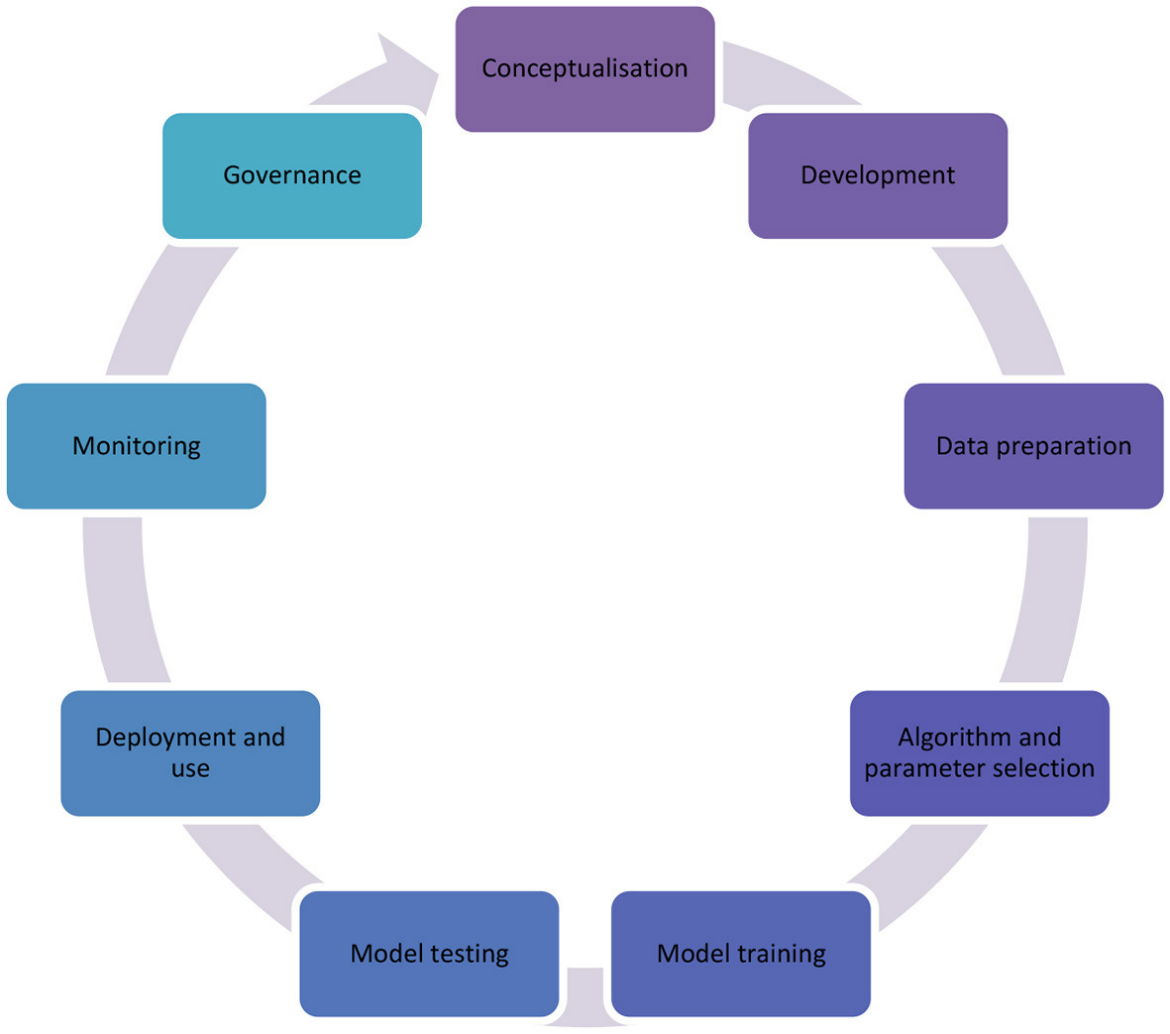
Phases in the AI lifecycle.

The TAC framework is seen as a scale, where transplantation is the least localized form of using models and creation is the most localized approach. When using the framework to analyze a model's appropriateness in the context of localization, several indicators can be considered, as seen in Table 3. The objective of using three separate categories in this framework is to encourage reflection in situations that could be nuanced, especially given the large amount of open-source software and common technologies that are used, even in localized contexts. Although we begin by examining each category more broadly, when it comes to implementation, we use it separately for each stage of the AI lifecycle, illustrated in Figure 1. That is, for any given AI system, some parts might be transplanted, others adapted, and other locally created. Table 4 shows the minimum and maximum scoring for each one of the possible indicators.

**Table 3:** Operationalizing the TAC framework.

**Dimension**	**Original source of development**	**Awareness of original developers**	**Ability to make modifications**	**Appropriateness for local context**
Transplantation	Outside target country	None to low	None to low	None
Adaptation	Outside target country	None to low	High	Medium to high
Creation	Inside target country	High	High	High

**Table 4:** TAC scoring coding manual.

**Lifecycle step/dimension**	**Transplantation (min score)**	**Adaptation**	**Creation (max score)**
Conceptualization	1	2	3
Data preparation	1	2	3
Algorithm and parameter selection	1	2	3
Model training	1	2	3
Model testing	1	2	3
Deployment and use	1	2	3
Monitoring	1	2	3
Governance	1	2	3

### 4.1. Transplantation

Transplantation is an approach that has often been used in models. We can consider, for example, a wide range of artificial intelligence models that were originally developed in the United States and were then used in other countries. Examples include social media platforms, who operate under a nearly uniform algorithm no matter what the target country.^1^ The Twitter, Facebook, YouTube and WhatsApp social media platforms were all developed within a 30-mile radius near San Francisco, California. Their user base covers the planet, with one-third of the world population currently active on Facebook, who also owns WhatsApp. Many features of the Facebook platform have been critiqued as overly centralized, including its hate-speech content moderation functionality, which is much better organized in English than in other languages (Waterson and Milmo, 2022).

Clashes between the intent of the technology and the context in which it was transplanted were apparent early in the adoption of the platforms. During the Arab Spring, for example, citizens in Tunisia and Egypt used the Facebook and Twitter platforms to organize pro-democracy protests, going against the interests of the companies themselves, who, as Tufecki (2017) reports, at best tolerated their activities, and at worst actively hindered them.

The economic policies discussed in section 1.2. can also be seen as examples of transplantation. They were largely developed by economists based at the IMF and World Bank headquarters in the United States who appeared to lack contextual knowledge of the country. Loan recipients found themselves unable to make modifications to the programs as developed and, as we have seen, suffered grave economic consequences from their lack of appropriateness.

### 4.2. Adaptation

Adaptation has increased in popularity with the rise of open-source technologies, which have allowed models developed in any location to be modified and reappropriated in a new context. This involves taking already pre-developed software and modifying it to fit a local context. The original software may have been developed by developers outside of the country who had low awareness of where this software would eventually be deployed, but has the ability to be modified by local developers.

In the case of adaptation, there is a certain nuance, in that all of AI is developed using some level of open-source software, from the programming language to the algorithms, to even, in some cases, open-source pre-trained models. Therefore, the distinction between adaptation and creation may be blurred in some cases. However, we maintain a difference in our framework in order to stimulate discussion and thought about the level of local development and ownership that is possible in AI projects. From the perspective of our framework, we present Adaptation as slightly less desirable than Creation, in that there are certain risks to keep in mind, namely when using pre-trained models or open-source datasets that could contain biases.

An example of this is the Mara smartphone company, which is based in Rwanda, and which uses the open-source Android software developed by Google. Another example was the adaptation of pre-trained NLP models to local languages in African news translation (Alabi et al., 2022).

### 4.3. Creation

Finally, creation involves the development of models directly in the target countries. In this case, the stage of the AI system lifecycle was developed completely locally, without the use of pre-trained models or external datasets. For example, SAFE Nigeria, an organization that fights violence against women, has developed several artificial intelligence-based technologies aimed to increase the safety of women. The important point about models being in the Creation phase is that they are originally developed in the target country, making them more likely to be appropriate to the setting in which they are used. It also has important implications in terms of data ownership and model governance, as we will see.

### 4.4. TAC scoring

Table 2 gives us a method for evaluating the dimension of any phase in the AI lifecycle. In the table below, we show how a scoring can be provided to each phase based on the selected dimension. For example, a project could have a score of 0 for Conceptualization if it was originally developed in the United States but is deployed in Senegal. However, it could have a 2 in Testing if the test protocol was originated in Senegal and implemented locally. In this way, a score from 0 to 2 can be selected for each phase of the lifecycle. The table below represents the coding manual used for scoring, which will be applied in Section 5.

## 5. Applications of the TAC framework in Canada, Libya and South Africa

**Figure 2:**
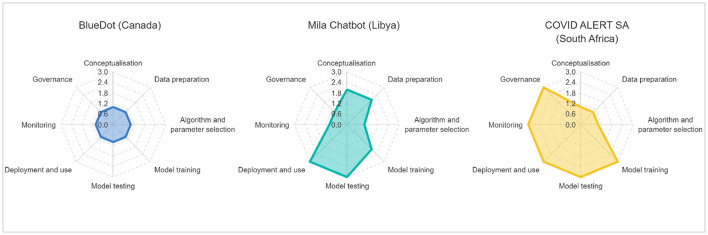
Estimated TAC score for each case study.

In this section, we will apply the above framework in three very short case studies, to illustrate the model in action in different contexts. We have selected the case studies for their diversity, applicability to localization principles, and the public availability of information regarding them. Figure 2 shows the preliminary TAC scores for each of the cases described.

### 5.1. BlueDot COVID-19 spread model (Canada)

BlueDot is a company founded by Kamran Khan, professor of medicine and public health at the University of Toronto (Stieg, 2020). The software company was started after the SARS outbreak of 2003, in order to better anticipate similar viruses. Like most COVID-19 spread models, it scores low on the TAC score, and is a strong contender for Transplantation. This is because the intention of the software is to scale quickly globally and to be managed centrally. At the time of writing, the software appears entirely managed from the Toronto office, using globally available data, such as airline ticketing data, and monitoring in 65 languages (we assume this might be based on publicly available social media and search data) (Stieg, 2020). While governments globally are invited to use the software, there is currently no information about localization of any of the phases of the AI development lifecycle. As such, this application obtains a minimum score for each phase.

### 5.2. Mila chatbot for COVID-19 (Libya)

As a different example, the Mila chatbot for COVID-19 was developed by the local World Food Programme (WFP) office (Fenton, 2021), in order to provide humanitarian aid information in English and Arabic during the pandemic. In an interview, the developers discuss in detail the differences between development in Libya and development in Iraq, explaining that the WFP country focal points are critical in ensuring that the chatbots are culturally appropriate (Fenton, 2021). The chatbot also appears to have been used for the current Ukraine crisis (Matak, 2022), to similarly communicate with aid beneficiaries during the humanitarian response. Just like the previous example, there has been an effort to scale the software for other applications beyond the initial case, although the scaling has taken place more slowly. Based on the publicly available articles, there also appears to have been an effort to address local differences and to integrate local focal points in the different phases of the project. In this case, we argue that the Mila chatbot is mostly an example of Adaptation.

### 5.3. COVID alert SA app (South Africa)

Finally, we examine the COVID-19 contact tracing application developed by the Government of South Africa. Like many countries around the world, South Africa based its contact tracing application on Google and Apple's exposure notification framework (Apple, 2022), which allowed persons to be notified if they had been within a certain distance of another person who had voluntarily declared that they had COVID-19. This framework used Bluetooth and did not store or share any personally identifiable information with the government. Based on available documentation, while the original framework was developed by these software companies, the South African government adapted it using its own developers. Aside from making the code open source, Google and Apple did not further contribute to the deployment of the applications in South Africa or anywhere else. However, Albertus and Makoza (2022) attributed low adoption rate of the application with privacy and data governance concerns, along with human rights issues. Albertus and Makoza (2022) also listed a number of technological and process challenges that made it difficult for users in South Africa to use, for example the enablement of Bluetooth draining the battery of older phones and the difficulty in entering a positive diagnosis with a health code. In terms of the TAC Framework, we nevertheless give a minimum score for the phases between conceptualization and algorithm and parameter selection, after which the South African government took control of the development process, and the phases receive a maximum score.

## 6. Methods and limitations for localizing models

Here, we discuss the methods for localizing AI for public health models, as well as limitations. We propose different methods for improving the localization of models, while also acknowledging that there are many cases where this might prove quite difficult. These are summarized in Table 5 below.

**Table 5:** Key principles of model localization.

**Objective**	**Principle**	**Description**
Transplantation to adaptation	Transparency and open source	Provide open source and transparent models so that those that want to adapt them to local contexts can do so.
	Transfer of ownership	Transfer model ownership to target country so that it can take charge of any future modifications.
Adaptation to creation	Capacity building	Increase model-building capacity, focusing on talent creation and retention.
	Long-term financing	Generate financing opportunities that are long-term, allowing for the development and staffing of research centers in the Global South.
Creation from the start	Decentralization	Ensure that the investment incentives in models are decentralized and promote both research and investment in Global South countries.
	Pluralism	Ensure that developers of the models come represent a variety of backgrounds, such as gender, ethnicity, nationality, ability, and so on.

### 6.1. Transitioning from transplantation to adaptation

As we have seen, the main difference between Transplantation and Adaptation of models is the lack of capacity of the target country to modify the model. In the case of economic models developed by the IMF, this was due to a lack of willingness by the model creator to allow the target country to make changes. We observe a similar behavior with algorithms used by social media platforms, where the models themselves are closely guarded and cannot be modified by the users. Moving from Transplantation to Adaptation therefore involves two principles: the sharing of the content of the model and the transfer of ownership of the model.

- *Transparency and open source*: In order to be adapted in a local context, components of AI systems, such as datasets, models and frameworks, must not only be open-source, but also explainable. This means that these components must have been developed with the intention of shareability, and with the possibility of localization by design.
- *Transfer of ownership*: Additionally, ownership of the model should be transferred. An important barrier to this exists in proprietary software, which can be used and sometimes integrated to other software, but which remains opaque and centrally managed.
- *Limitations*: From a legal perspective, intellectual property laws can be an obstacle, where local ownership is not desired or possible. In addition, local technical skill must be available to adapt these models, as well as maintain the AI systems throughout their deployment and use.

### 6.2. Transitioning from adaptation to creation

The key differences between Adaptation and Creation involve the source of the model and the awareness of the developers. In the Adaptation phase, the original model is developed outside of the target country, and the original development team are likely to lack awareness of the context of the target country.

- *Capacity building*: A key component to the local creation of AI systems remains local capacity building. While there has been significant investment, over the last few years, in AI skills development in the Global South, countries face challenges such as exportation of local talent (also called the brain drain), local infrastructure and resources.
- *Long-term financing*: Similarly, efforts to support capacity building in the Global South can be short-term, while the development of a local AI ecosystem requires funding and stability over many years, or even decades.
- *Limitations*: The capacity to create depends also on the states themselves and their cultures, finding a balance between taking good cooperation between states into account and also certain specificities, such as local ecosystems and traditional knowledge.

### 6.3. Promoting creation and cooperation in model development

Finally, when promoting the creation at the beginning of the model development process, we can consider two more principles: decentralization and pluralism.

- *Decentralization*: Efforts to promote local model development, whether it is AI model, legal model, or any other model, will require a rebalancing of geopolitical power and resources. From an AI perspective, this does mean that efforts to provide local alternatives to large systems such as social media platforms, or require interoperability between systems, will be prioritized.
- *Pluralism*: Similarly, recent technological developments have suffered from a lack of consideration of pluralism and diversity. Many of the current solutions are unilingual, and developed with a specific cultural context in mind. Efforts toward localization will therefore also need to incorporate the pluralist mindset, in which there are many different approaches and perspectives possible.

The table below shows the key principles in localizing models as discussed above.

## 7. Conclusion

In this paper, we therefore examined the historical and geopolitical tendencies toward centralizing policy models. We argued that this could have particularly detrimental effects in public health, where local considerations can be extremely relevant to population health. We proposed that analyzing models through a localization lens would therefore help us to identify areas where localization could be appropriate and attainable. We therefore presented the TAC Framework, which aims to provide a straightforward method for analyzing a given project involving AI for public health policy.

Many of the concepts presented here could be applied to AI in other contexts. In fact, the issue of localization in AI for public policy is an important one, which has still not been extensively explored in research. It is our hope that researchers and policymakers will seek to apply the TAC framework to different scenarios in order to continue to add nuance and improvements.

The use of AI for public health policy will likely continue to increase in the coming years, and challenges and best practices will need to continuously be revisited. The development of models, AI or otherwise, can certainly be exciting, but still requires caution and reflection to make sure the these are adapted to the reality they attempt to represent. Fundamentally, the objective of the TAC Framework is to encourage a pause in the modeling process, to allow for the consideration of more appropriate and relevant development approaches.

## Author contributions

The author confirms being the sole contributor of this work and has approved it for publication.

## Footnotes

^1^There are some small variations per country, specifically for filtering hate speech or political dissidents, particularly in the case of Facebook.

## References

[B1] Alabi J. O. Adelani D. I. Mosbach M. Klakow D. (2022). “Adapting pre-trained language models to african languages via multilingual adaptive fine-tuning,” in Proceedings of the 29th International Conference on Computational Linguistics, 4336–4349.

[B2] Albertus R. W. Makoza F. (2022). An analysis of the COVID-19 contact tracing App in South Africa: challenges experienced by users. African J. Sci. Technol. Innov. Dev. 15, 124–134. 10.1080/20421338.2022.2043808

[B3] Alsdurf H. Belliveau E. Bengio Y. Deleu T. Gupta P. Ippolito D.. (2020). Covi white paper. arXiv.

[B4] Apple (2022). Exposure Notification. Developer Platform. Available online at: https://developer.apple.com/documentation/exposurenotification (accessed August 06, 2023).

[B5] Arnstein S. R. (1969). A Ladder of Citizen Participation. JAIP. 35, 216–224. 10.1080/01944366908977225

[B6] Battikha A. M. (2002). Structural Adjustment and the Environment: Impacts of the World Bank and IMF Conditional Loans on Developing Countries.

[B7] Blaise L. (2020). “En Tunisie, un chatbot pour contrer le coronavirus,” in Le Monde. Available online at: https://www.lemonde.fr/afrique/article/2020/06/02/en-tunisie-un-chatbot-pour-contrer-le-coronavirus_6041546_3212.html (accessed August 06, 2023).

[B8] Buolamwini J. Gebru T. (2018). “Gender shades: Intersectional accuracy disparities in commercial gender classification,” in Paper Presented at the Conference on Fairness, Accountability and Transparency.

[B9] Calzati S. (2021). Decolonising ‘data colonialism' propositions for investigating the realpolitik of today's networked ecology. Television New Media 22, 8. 10.1177/1527476420957267

[B10] Cook K. (2020). The Psychology Of Silicon Valley: Ethical Threats and Emotional Unintelligence in the Tech Industry. Cham: Springer Nature, 314. 10.1007/978-3-030-27364-4

[B11] Cooke B. (2003). A new continuity with colonial administration: participation in development management. Third World Quart. 24, 47–61. 10.1080/713701371

[B12] Couldry N. Mejias U. (2018). Data Colonialism: Rethinking Big Data's Relation to the Contemporary Subject. Television New Media 20, 336–349. 10.1177/1527476418796632

[B13] Dieye A. (2020). “Sustainability of the senegal socioeconomic model,” in An Islamic Model for Stabilization and Growth. London: Palgrave Macmillan, 157–182. 10.1007/978-3-030-48763-8_6

[B14] Easterly W. (2003). “IMF and World Bank structural adjustment programs and poverty,” in Managing Currency Crises in Emerging Markets. Chicago: University of Chicago Press, 361–392. 10.7208/chicago/9780226155425.003.0012

[B15] Fenton S. (2021). “A chatbot named Mila: Answering the call for people in Libya,” in WFP. Available online at: https://www.wfp.org/stories/chatbot-named-mila-answering-call-people-libya (accessed August 06, 2023).

[B16] Foley T. J. (2022). The Judicial Failsafe: American legal colonialism in the Philippines. Am. J. Legal Hist. 62, 158–181. 10.1093/ajlh/njac009

[B17] Fournier-Tombs E. Castets-Renard C. (2022). “Algorithmes et propagation de normes culturelles sexospécifiques (Algorithms and the propagation of gendered cultural norms),” in Forthcoming in: IA, Culture et Médias. Presses de l'Université Laval, Collection Éthique, IA et Sociétés de l'OBVIA.

[B18] Geddes L. (2022). “Predicting a pandemic: how AI helped predict COVID-19′s twists and turns,” in GAVI. Available online at: https://www.gavi.org/vaccineswork/predicting-pandemic-how-ai-helped-predict-covid-19s-twists-and-turns (accessed August 06, 2023).

[B19] Gómez-Ramírez O. Medeiros P. Wainer R. Iyamu I. (2021). 13 Does the ‘Canada COVID-19 Alert' App Stand up to Critical Scrutiny? A Rapid Qualitative Assessment.

[B20] Heaven D. (2022). “Hundreds of AI tools have been built to catch COVID. None of them helped,” in MIT Technology Review. Available online at: https://www.technologyreview.com/2021/07/30/1030329/machine-learning-ai-failed-covid-hospital-diagnosis-pandemic/ (accessed August 06, 2023).

[B21] Imperial College (2022). COVID-19 Planning Tools. Available online at: https://www.imperial.ac.uk/mrc-global-infectious-disease-analysis/disease-areas/covid-19/covid-19-planning-tools/ (accessed August 06, 2023).

[B22] Ismi A. (2004). Impoverishing a Continent: The World Bank and the IMF in Africa. Ottawa: Canadian Centre for Policy Alternatives.

[B23] King I. (2022). “True Sovereignty? The CFA Franc and French Influence in West and Central Africa,” in Harvard International Review. Available online at: https://hir.harvard.edu/true-sovereignty-the-cfa-franc-and-french-influence-in-west-and-central-africa/ (accessed August 06, 2023).

[B24] Luengo-Oroz M. Bullock J. Pham K. H. Lam C. S. N. Luccioni A. (2021). From artificial intelligence bias to inequality in the time of COVID-19. IEEE Technol. Soc. Magaz. 40, 71–79. 10.1109/MTS.2021.3056282

[B25] Matak V. (2022). “Humanitarian chatbot: How tech bridges gap between people and the assistance they need in Ukraine,” in WFP. Available online at: https://www.wfp.org/stories/humanitarian-chatbot-how-tech-bridges-gap-between-people-and-assistance-they-need-ukraine (accessed August 06, 2023).

[B26] Mazatlan Post (2019). Teaching the Mayan Language will be mandatory in Yucatan Schools. Available online at: https://themazatlanpost.com/2019/12/09/teaching-the-mayan-language-will-be-mandatory-in-yucatan-schools/ (accessed August 06, 2023).

[B27] Mbow C. Mertz O. Diouf A. Rasmussen K. Reenberg A. (2008). The history of environmental change and adaptation in eastern Saloum–Senegal—Driving forces and perceptions. Global Planet. Chang. 64, 210–221. 10.1016/j.gloplacha.2008.09.00826056532

[B28] Merriam Webster (2022). “Model,” in Dictionary Entry. Available at: https://www.merriam-webster.com/dictionary/model

[B29] Merry S. E. (1991). Law and colonialism. Law Soc. Rev. 25, 889–922. 10.2307/3053874

[B30] Milan S. Treré E. (2019). Big data from the south(s): beyond data universalism. Television New Media 20, 319–335. 10.1177/1527476419837739

[B31] Ministère de l'Europe et des Affaires Étrangères (MEAE) Gouvernement Français. (2022). France Diplomacy – Senegal. Available online at: https://www.diplomatie.gouv.fr/en/country-files/senegal/ (accessed August 06, 2023).

[B32] Mitchell E. (2016). “How silicon valley's palantir wired Washington,” in Politico. Available online at: https://www.politico.com/story/2016/08/palantir-defense-contracts-lobbyists-226969 (accessed August 06, 2023).

[B33] Mohamed S. Png M. Isaac W. (2020). Decolonial AI: decolonial theory as sociotechnical foresight in artificial intelligence. arXiv. 10.1007/s13347-020-00405-8

[B34] Moore M. Tambini D. (2018). Digital dominance: the power of Google, Amazon, Facebook, and Apple. Oxford: Oxford University Press.

[B35] Nguyen D. Q. Vo N. Q. Nguyen T. T. Nguyen-An K. Nguyen Q. H. Tran D. N.. (2022). BeCaked: an explainable artificial intelligence model for COVID-19 forecasting. Scientific Rep. 12, 1–26. 10.1038/s41598-022-11693-9 35562369 PMC9105619

[B36] Norton Rose Fulbright (2020). Contact Tracing Apps: a New World for Data Privacy. Available online at: https://www.nortonrosefulbright.com/en-cn/knowledge/publications/d7a9a296/contact-tracing-apps-a-new-world-for-data-privacy (accessed August 06, 2023).

[B37] Ollivier T. (2020). “Au Sénégal, un robot au chevet des patients atteints du coronavirus,” in Le Monde. Available online at: https://www.lemonde.fr/afrique/article/2020/05/26/au-senegal-un-robot-au-chevet-des-patients-atteints-du-coronavirus_6040836_3212.html (accessed August 06, 2023).

[B38] Park S. Humphry J. (2019). Exclusion by design: intersections of social, digital and data exclusion. Informat. Commun. Soc. 22, 934–953. 10.1080/1369118X.2019.1606266

[B39] Pohle J. Thiel T. (2021). “Digital sovereignty,” in Practicing Sovereignty: Digital Involvement in Times of Crises, eds H. B. Irrgang, D. Joost, and A. Gesche Untridig (Bielefeld: Verlag), 47–67.

[B40] Ricaurte P. (2019). Data epistemologies, the coloniality of power, and resistance. Television New Media 20, 4. 10.1177/1527476419831640

[B41] Roberts M. Driggs D. Thorpe M. Gilbey J. Yeung M. Ursprung S.. (2021). Common pitfalls and recommendations for using machine learning to detect and prognosticate for COVID-19 using chest radiographs and CT scans. Nat. Mach. Intell. 3, 199–217. 10.1038/s42256-021-00307-0

[B42] Sahbaz U. (2019). Artificial intelligence and the risk of new colonialism. Horizons 14, 58–71.

[B43] Shafiu R. M. (2021). “Role of IMF lending preconditions in Nigeria,” in IEOM Society Papers. Available online at: http://www.ieomsociety.org/singapore2021/papers/1205.pdf (accessed August 06, 2023).

[B44] Silcoff S. (2020). “Element AI sold for $230-million as founders saw value mostly wiped out, document reveals,” in The Global and Mail. Available online at: https://www.theglobeandmail.com/business/article-element-ai-sold-for-230-million-as-founders-saw-value-wiped-out/

[B45] Statista (2022a). Number of Monthly Active Facebook Users Worldwide. Available online at: https://www.statista.com/statistics/264810/number-of-monthly-active-facebook-users-worldwide/ (accessed August 06, 2023).

[B46] Statista (2022b). Instagram Number of Global Users. Available online at: https://www.statista.com/statistics/183585/instagram-number-of-global-users/ (accessed August 06, 2023).

[B47] Statista (2022c). WhatsApp Global Unique Users. Available online at: https://www.statista.com/statistics/1306022/whatsapp-global-unique-users/ (accessed August 06, 2023).

[B48] Stein H. (1992). Economic Policy and the IMF in Tanzania: Conditionality, Conflict and Convergence. Oxforshire: Routledge.

[B49] Stieg C. (2020). “How this Canadian Startup Spotted Coronavirus Before Anyone Else Knew About it,” in CNBC. Available online at: https://www.cnbc.com/2020/03/03/bluedot-used-artificial-intelligence-to-predict-coronavirus-spread.html (accessed August 06, 2023).

[B50] Stiglitz J. (2002). Globalization and Its Discontents. New York, NY: W. W. Norton.

[B51] Timmerman A. (2020). “Meet the trans woman behind taiwan's successful grassroots coronavirus initiatives,” in Vice. Available online at: https://www.vice.com/en/article/jge9jx/taiwan-trans-woman-minister-audrey-tang-coronavirus-tech (accessed August 06, 2023).

[B52] Tufecki Z. (2017). Twitter and Tear Gas. New Haven: The power and fragility of networked protest.

[B53] Tworek H. Beacock I. Ojo E. (2020). Democratic Health Communications during Covid-19: A RAPID Response. Vancouver, BC: University of British Columbia. Available online at: https://democracy2017.sites.olt.ubc.ca/files/2020/09/Democratic-Health-Communication-during-Covid_FINAL.pdf (accessed August 06, 2023).

[B54] University of Waterloo (2022). AI models identify COVID-19 patients at the greatest risk of death, injury. Available online at: https://uwaterloo.ca/news/media/ai-models-identify-covid-19-patients-greatest-risk-death (accessed August 06, 2023).

[B55] UNOCHA (2021). OCHA-Bucky Model to Inform Humanitarian Operations. Available online at: https://centre.humdata.org/ocha-bucky-a-covid-19-model-to-inform-humanitarian-operations/ (accessed August 06, 2023).

[B56] Waterson J. Milmo D. (2022). “Facebook whistleblower frances haugen calls for urgent external regulation,” in The Guardian.

[B57] York University. (2021). New AI Powered algorithm to predict third wave of COVID-19 in South Africa. Available online at: https://news.yorku.ca/2021/04/12/new-ai-powered-algorithm-to-predict-third-wave-of-covid-19-in-south-africa/ (accessed August 06, 2023).

[B58] Zakrezwski C. (2022). “Tech companies spent almost $70 million lobbying Washington in 2021 as Congress sought to rein in their power,” in Washington Post. Available online at: https://www.washingtonpost.com/technology/2022/01/21/tech-lobbying-in-washington/ (accessed August 06, 2023).

